# Evaluation of Methyl Bromide Alternatives Efficacy against Soil-Borne Pathogens, Nematodes and Soil Microbial Community

**DOI:** 10.1371/journal.pone.0117980

**Published:** 2015-02-27

**Authors:** Hongwei Xie, Dongdong Yan, Liangang Mao, Qiuxia Wang, Yuan Li, Canbin Ouyang, Meixia Guo, Aocheng Cao

**Affiliations:** 1 Institute of Plant Protection, Chinese Academy of Agricultural Sciences, Beijing, China; 2 State Key Laboratory for Biology of Plant Disease and Insect Pests, Beijing, China; Institute for Sustainable Plant Protection, C.N.R., ITALY

## Abstract

Methyl bromide (MB) and other alternatives were evaluated for suppression of Fusarium spp., *Phytophthora* spp., and *Meloidogyne* spp. and their influence on soil microbial communities. Both *Fusarium* spp. and *Phytophthora* spp. were significantly reduced by the MB (30.74 mg kg^-1^), methyl iodide (MI: 45.58 mg kg^-1^), metham sodium (MS: 53.92 mg kg^-1^) treatments. MS exhibited comparable effectiveness to MB in controlling *Meloidogyne* spp. and total nematodes, followed by MI at the tested rate. By contrast, sulfuryl fluoride (SF: 33.04 mg kg^-1^) and chloroform (CF: 23.68 mg kg^-1^) showed low efficacy in controlling *Fusarium* spp., *Phytophthora* spp., and *Meloidogyne* spp. MB, MI and MS significantly lowered the abundance of different microbial populations and microbial biomass in soil, whereas SF and CF had limited influence on them compared with the control. Diversity indices in Biolog studies decreased in response to fumigation, but no significant difference was found among treatments in PLFA studies. Principal component and cluster analyses of Biolog and PLFA data sets revealed that MB and MI treatments greatly influenced the soil microbial community functional and structural diversity compared with SF treatment. These results suggest that fumigants with high effectiveness in suppressing soil-borne disease could significantly influence soil microbial community.

## Introduction

*Fusarium oxysporum*, *Phytophthora infestans* and nematodes such as *Meloidogyne* spp. can cause great damage in greenhouse crops and consequently lead to reductions in crop yield. Methyl bromide (MB) is an effective fumigant against soil-borne pathogens and nematodes, however, because it depletes the ozone layer, methyl bromide will be phased out by 2015 in China. Thus, several alternatives such as metham sodium(MS), 1,3-dichloropropene (1,3-D), chloropicrin(Pic), sulfuryl fluoride(SF), methyl iodide(MI) and other substances have been studied as replacements for methyl bromide pre-plant soil treatment in agriculture [1–4]. MS has positive effects in controlling soil-borne fungal pathogens (e.g. *Verticillium dahliae*, *Fusarium oxysporum f*. *sp*. *Vasinfectum* and *Sclerotinia sclerotiorum*) [1,5], nematodes and weeds [6,7]. SF is inorganic and has a significantly higher vapor pressure and lower binding potential [8]. The efficacy of SF has been demonstrated against warehouse pests [9,10] and is used as an alternative to MB for stored products and structures. However, there are relatively few reports about its effect on soil-borne pathogens and nematodes. MI is reported to have comparable effects to MB [2,11]. In container trials, MI was significantly more effective than MB against the plant parasitic nematodes *Meloidogyne incognita*, *Heterodera schachtii* and *Tylenchulus semipenetrans* and the plant pathogenic fungus *Rhizoctonia solani* [2].

Soil microorganisms, composed of many taxa, play an important role in plant nutrient cycling, decomposition and mineralization. Previous studies on fumigants have mostly focused on their efficacies against pests, pathogens, weeds and environmental behavior [7,12,13]. However, the impact of fumigants on soil microflora is largely unknown [14]. Recently, several studies investigated the impact of MB, 1,3-D and Pic fumigation on microbial communities [15,16]. Tanaka [17] reported that the effects on microbial biomass and substrate utilization, assessed using the Biolog method, were larger in the chloropicrin and steam sterilization treatments than in the MB treatment. Supplementary studies on the dynamics of soil microbial communities after chloropicrin and steam sterilization treatments were performed by Yamamoto [18]. However, little research has been conducted on the influence of SF, MI and MS on soil microbial communities.

Ideally, a pesticide should be toxic only to the target organisms; however, fumigants are a class of pesticide with broad biocidal activity, affecting many non-target soil organisms [19]. Therefore, to gain more understanding of the impacts of fumigants on target and non-target soil microorganisms, our studies investigated the effects of methyl bromide and several alternatives on two soil-borne pathogens, nematodes, and soil microbial communities.

## Materials and Methods

### Soil type and microcosm study

Soil samples were collected from the top 15cm of soil in a greenhouse that produces tomato and cucumber in rotation, located in Tongzhou district, east of Beijing; GPS coordinates are 116°44’E, 39°54’N. Soil samples were taken with the authorization of the Institute of Tongzhou Agricultural Science (Beijing City), and no other specific permissions were required for the field site. The greenhouse had a history of nematode and soil-borne pathogen infestation. The soil is classified as a sandy loam, composed of 2.9% clay, 38.6% silt and 58.5% sand. The pH was 6.86, organic matter was 33.6 g kg^-1^, ammonium nitrogen was 73.0 mg kg^-1^, nitrate nitrogen was 249.5 mg kg^-1^, the rapidly-available potassium was 443.5 mg kg^-1^ and the available phosphorus was 657.7 mg kg^-1^. Field moist soil was passed through a 2-mm sieve. Soil samples were mixed and the moisture was adjusted to 15% in order to ensure optimal fumigant distribution. Soil samples were stored at room temperature for 48 h before being used in the experiment.

Soil samples were placed in microcosms consisting of glass jars containing 1 kg (dry weight) soil. Jars were sealed with an air-tight lid and the fumigants were added by a syringe as freshly prepared aqueous solutions sufficient to bring soil moisture content to field capacity. The experiment design consisted of four fumigants and a control in three replicated microcosms. In order to investigate the effectiveness of CF in room temperature and normal pressure, a treatment of CF was also performed in the experiment (Table 1). The treatments were tested according to the rates in Table 1. The microcosms were sealed for 7d after fumigant application and were vented continuously through a small opening on the cover. Soil samples were blended thoroughly, collected after fumigation, and stored at 4°C and -20°C independently for microbial community analysis.

**Table 1:** Fumigant properties, supplier and concentration used in the experiment.

Soil fumigant	Supplier	Percent a.i^a^	Density kg·m^-3^ (25°C)	Concentration mg·kg^-1^ soil (dry weight)
Methyl bromide	Jianxing Chemical Co. Ltd, Zhejiang province, China	98%	3.8741	30.74
Sulfuryl fluoride	Liming Chemical Co. Ltd, Zhejiang province, China	99.8%	4.1646	33.04
Methyl iodide	Sinopharm Chemical Reagent Beijing Co. Ltd, Beijing, China	100%	2.28	45.58
Metham sodium	Fengshou Pesticide Co. Ltd, Shenyang province, China	42%	0.84246	53.92
Chloroform	Sinopharm Chemical Reagent Beijing Co. Ltd, Beijing, China	100%	1.5	23.68
Untreated control				

^a^ active ingredient.

### Survival of soil-borne pathogens *Fusarium* spp. and *Phytophthora* spp.

Soil samples were mixed thoroughly after fumigation and air dried; 10 g sub-samples was collected and added to 90mL sterilized water, and shaked for 30 min before use.The number of *Fusarium* spp. propagules in each sample was determined by dilution plating on modified Komada’s medium [20] on three replicated plates. The number of *Phytophthora* spp. propagules in each sample was determined by dilution plating on modified Masago’s medium [21] on three replicated plates. Plates were incubated in darkness at 28°C for 4d. Counts from the three replicates were combined to obtain mean values.

### Survival of root knot nematodes

Soil samples were collected after 7d fumigation. Nematodes were separated and counted in 100g soil using a standard sieving and centrifugation procedure [22]. In addition, the identification and counting of second stage juveniles of *Meloidogyne* spp. was confirmed by microscope.

### Survival of fungi, bacteria and actinobacteria on agar plates

10 g soil samples was collected and added to 90mL sterilized water, and shaked for 30 min. Soil suspensions were diluted in 10^-2^, 10^-3^ and 10^-4^ for fungi, bacteria and actinobacteria studies, respectively. Rose bengal medium, beef extract-peptone and Gauze's medium No.1 were used for fungi, bacteria and actinobacteria isolation, respectively.

### Influence of treatments on soil microbial biomass carbon and nitrogen

Moist 40g soil samples were divided into two 20g subsamples. The non-fumigated control samples were extracted immediately with 80mL 0.5M K_2_SO_4_ (extractant-to-soil ratio of 4:1) for 30 min by shaking under 200 rpm. For the fumigation treatments, 50-mL glass vials containing 20g subsamples were placed into a desiccator containing 25mL ethanol-free CHCl_3_ and a few boiling chips and the desiccator was evacuated until the CHCl_3_ had boiled vigorously for 5 min. The desiccator was incubated in the dark at 25°C for 24h. After fumigation, CHCl_3_ was removed by repeated (six-fold) evacuations and extracted with 0.5M K_2_SO_4_ as described above. The soil microbial biomass carbon and nitrogen were evaluated by dichromate oxidation method and ninhydrin-reactive nitrogen method, respectively [23].

### Changes in community-level carbon sources utilization by Biolog method

The Biolog ECO Microplates (Biolog Inc., Hayward, CA) contains three replicated wells of 31 carbon substrates which are predominantly amino acids (*n* = 6), carbohydrates (*n* = 10), and carboxylic acids (*n* = 7). Ten-fold serial 0.85% NaCl dilutions of the soil samples were prepared and 150 μ of the 10^-3^ dilutions were inoculated on ECO microplates. The plates were incubated in the dark at 25°C immediately (0h) and every 24h, and quantified in λ = 590 nm by measuring well absorbance using an automated microplate reader (BioTek Instruments, Inc., USA).

### Soil microbial community structural diversity studied by phospholipid fatty acid

Phospholipid fatty acid (PLFA) analysis was based on Bossio [24] and Frostegård [25] as described by Zhang [26]. Each 5 gram soil sample was freeze-dried and extracted using a one-phase mixture of CHCl_3_/methanol/citric acid buffer (0.15 M, pH 4) (1:2:0.8, v/v/v). The lower CHCl_3_ layer was then collected and dried under N_2_ for lipid fractionation after being shaked and centrifuged. Afterwards, the extracted lipids were separated by silica gel columns (Agela, Inc.) into glycolipids, neutral lipids, and polar lipids. The polar lipids were then transesterified with methanolic KOH to recover the PLFAs as methyl esters through methanolysis in hexane. Finally, the hexane supernatant containing the resultant fatty acid methyl esters (FAMEs) was separated, quantified and identified by gas chromatography mass spectrometry (GC–MS). A PolarisQ ion-trap GC–MS (Thermo Fisher Scientific, Inc.) with a HP-5 ms column (60 m × 0.25 mm inner diameter, 0.25 μm film thickness) was used for FAME identification. The microbial biomass was assessed by 30 fatty acids. The PLFAs 18:1ω9t, 18:1ω9c, 18:2ω6c were used as indicators of fungi [25], while 10Me18:0 and 10Me17:0 were used to indicate actinobacteria [27]. The total PLFAs of cy19:0, 18:1ω9t, 18:1ω9c, cy17:0, i17:0, 16:1ω9c, i16:0, a15:0, i15:0, 14:1ω5 and 20:1ω9c were chosen to represent the bacteria biomass. Gram-positive (G^+^) bacteria were determined by the sum of branched phospholipids i15:0, a15:0, i16:0 and i17:0, whereas the PLFAs cy19:0, 18:1ω9t, 18:1ω9c, cy17:0 and 16:1ω9c were indicative of Gram-negative (G^-^) bacteria.

Prior to being subjected to principal component analysis (PCA) and cluster analysis, the results were expressed as a percentage of the total PLFA.

### Data analysis

The average well color development (AWCD) values which collected in 96h after inoculation were subjected to PCA and cluster analysis. Shannon Index, McIntosh Index and Simpson Index were calculated according to the formulas described by Staddon [28].

Statistics were calculated using SPSS 16.0 for Windows software (SPSS Inc). The effects of different fumigation treatments were examined using ANOVA, and significant differences were accepted at p < 0.05. All values reported are the means (SE) of three replicates, except for data which was used in Biolog and PLFA PCA and cluster analyses.

Cluster analyses were conducted in Biolog and PLFA studies using Hierarchical clustering according to the Ward-method.

## Results

### Survival of soil-borne pathogens and nematodes

Overall, the MB, MI and MS treatments had somewhat similar effects and showed greater effectiveness in suppressing soil-borne pathogens and nematodes compared with SF and CF treatments at the tested rates (Table 2). As an alternative to MB, MS showed great efficacy in controlling *Fusarium* spp. and *Meloidogyne* spp., while SF showed little effect in controlling soil-borne pathogens and nematodes. CF showed no significant difference with untreated control in controlling *Phytophthora* spp. and *Meloidogyne* spp..

**Table 2:** Effect of treatments on soil-borne pathogens, total soil nematodes and *Meloidogyne* spp. population.

Treatment^a^	Soil-borne pathogens (CFU^c^ g^-1^)		% reduction in nematodes (100g^-1^soil)^d^	
	*Fusarium* spp.	*Phytophthora* spp.	Total	*Meloidogyne* spp.
MB	849 (4.4) a^b^	1311 (47.0) a	96.59 (0.9) a	97.11 (1.7) a
SF	1102 (4.4) b	1858 (90.2) b	48.91 (2.5) c	39.66 (4.5) c
MI	804 (39.5) a	1064 (40.1) a	92.80 (0.9) b	81.31 (4.1) b
MS	695 (46.6) a	1289 (17.8) a	92.49 (0.3) b	91.53 (3.3) a
CF	1256(79.1)c	1991(1.2)bc	31.59(2.0)d	24.83 (2.3) cd
Untreated control	1789 (64.1) d	2200 (1.2) c	7.65 (1.3) e	5.16 (1.1) d

Data are shown as the arithmetic mean with standard error (*n* = 3).

^a^ MB: methyl bromide, SF: sulfuryl fluoride, MI: methyl iodide, MS: metham sodium, CF: chloroform.

^b^ Data in the same column followed by the same letter are not significantly different at the P<0.05 level.

^c^ CFU: colony-forming unit.

^d^ Reductions in nematodes are shown as percentages.

### Influence of fumigants on soil microbial communities and their ratios in the soil

Agar plate observations and PLFA analysis both indicated that MB, MI and MS treatments significantly reduced the fungi, bacteria, G^+^ bacteria, G^-^ bacteria and actinobacteria populations in soil. In keeping with their impact on pathogens and nematodes, both the SF and CF treatment had only mild influences on soil culturable fungi, actinobacteria and bacteria populations (Table 3). Although MB, MI and MS greatly influenced the abundance of soil microbial communities, there was no significant difference among the fumigants treatments and control on the ratios of G^+^/G^-^ and Fungi/ Bacteria.

**Table 3:** Influence of treatments on soil microbial communities and their ratios.

Treatment^a^	Culturable Microbes in Agar Plates			PLFA Analysis						
	Fungi (×10^5^CFU^c^ g^-1^)	Bacteria (×10^8^CFU g^-1^)	Actinobacteria (×10^7^CFU g^-1^)	Fungi (nmol g^-1^)	Bacteria (nmol g^-1^)	Actinobacteria (nmol g^-1^)	G^+^ (nmol g^-1^)	G^-^ (nmol g^-1^)	G^+^/ G^-^	Fungi/ Bacteria
MB	1.80 (0.1) a^b^	1.96 (0.1) a	4.54 (0.6) b	19.60 (2.4) a	47.95 (4.7) a	7.55 (1.3) a	20.06 (2.2) a^b^	25.95 (2.8) a	1.30 (0.1) a	2.47 (0.1) b
SF	2.40(0.1)b	4.35 (0.2) b	7.58 (0.6) c	43.25 (1.0) c	93.31 (4.1) c	16.85 (1.5) c	35.48 (2.4) c	53.75 (1.8) c	1.52 (0.1) bc	2.16 (0.1) a
MI	1.78(0.2)a	2.30 (0.1) a	0.65 (0.1) a	28.20 (0.4) b	61.53 (1.2) b	9.38 (0.3) ab	23.52 (1.1) ab	36.19 (0.5) b	1.54 (0.7) bc	2.18 (0.1) a
MS	1.84(0.1)a	2.04 (0.1) a	5.78 (0.2) bc	31.41 (2.4) b	68.11 (4.2) b	12.32 (1.0) b	24.97 (1.9) b	40.57 (2.4) b	1.63 (0.02) c	2.17 (0.03) a
CF	2.39(0.1)b	4.29 (0.4) b	7.54 (1.0) cd	46.02 (3.0) c	97.66 (6.1) c	16.62 (1.2) c	35.30 (1.9) c	57.31 (3.8) c	1.62 (0.03) bc	2.12 (0.02) a
Untreated control	3.66(0.3)c	6.35 (0.5) c	8.80 (0.4) cd	60.01 (1.3) d	137.41 (3.6) d	23.54 (0.2) d	54.56 (3.2) d	76.77 (0.8) d	1.42 (0.1) ab	2.29 (0.1) ab

Data are shown as the arithmetic mean with standard error (*n* = 3).

^a^ MB: methyl bromide, SF: sulfuryl fluoride, MI: methyl iodide, MS: metham sodium, CF: chloroform.

^b^ Data in the same column followed by the same letter are not significantly different at the P<0.05 level.

^c^ CFU: colony-forming unit.

### Parameters of soil microbial biomass in response to fumigation

Soil microbial biomass carbon and nitrogen studies indicated that MB, MI and MS significantly reduced the soil microbial biomass whereas SF and CF just had mild influence on them (Table 4). In PLFA studies, MB greatly reduced the total PLFA values which indicate the living microbial biomass followed by MI and MS, SF and CF had equivalent impacts on total PLFA values.

**Table 4:** Parameters of soil microbial biomass in response to fumigation.

Treatment^a^	Soil microbial biomass carbon (mg C kg^-1^soil)	Soil microbial biomass nitrogen(mg N kg^-1^soil)	Total PLFA(nmol g^-1^)
MB	9.69 (0.1) a	8.80 (0.2) ab	146 (9.0) a
SF	74.24 (6.5) c	22.26 (0.6) c	228 (5.3) cd
MI	12.78 (3.2) ab	7.04 (1.6) a	170 (11.3) b
MS	22.31 (3.3) b	11.14 (0.6) b	206 (9.5) c
CF	50.55 (23.1) bc	25.90 (2.2) cd	245 (14.4) d
Untreated control	70.49 (6.3) c	32.15 (1.9) d	337 (5.0) e

Data are shown as the arithmetic mean with standard error (*n* = 3).

^a^ MB: methyl bromide, SF: sulfuryl fluoride, MI: methyl iodide, MS: metham sodium, CF: chloroform.

Data in the same column followed by the same letter are not significantly different at the P<0.05 level.

### Soil microbial community diversity studies

In Biolog studies, there was no significant difference between MB and MI treatments in Shannon, McIntosh and Simpson diversity indices (Table 5), all the diversity values of MB, MI and MS treatments were significantly lower than other treatments. SF treatment had the highest diversity values (Shannon, McIntosh and Simpson diversity value) compared with other treatments. Although significant difference happened on functional diversity indices among the treatments in Biolog studies, there was no significant difference in the Shannon values among different treatments while refer to PLFA studies. The results indicated that soil microbial structural diversity dose not change when soil microbial functional diversity changed.

**Table 5:** Diversity indices for fumigation treatment.

Treatment ^a^	Biolog Analysis			PLFA Analysis
	Shannon Diversity Index	McIntosh Diversity Index	Simpson Diversity Index	Shannon Diversity Index
MB	2.85 (0.01) a^b^	3.57 (0.33) a	15.17 (0.07) a	2.88 (0.00) ab
SF	3.20 (0.04) c	7.26 (0.56) d	23.27 (0.32) c	2.85 (0.01) a
MI	2.81 (0.02) a	4.19 (0.25) a	14.61 (0.38) a	2.91 (0.03) b
MS	3.01 (0.02) b	4.16 (0.24) a	18.04 (0.68)b	2.89 (0.03) ab
CF	2.94 (0.04) b	5.30 (0.05) bc	16.65 (0.78) b	2.87 (0.01) ab
Untreated control	3.19 (0.03) c	6.31 (0.11) cd	22.79 (0.52) c	2.89 (0.01) ab

Data are shown as the arithmetic mean with standard error (*n* = 3).

^a^ MB: methyl bromide, SF: sulfuryl fluoride, MI: methyl iodide, MS: metham sodium, CF: chloroform.

^b^ Data in the same column followed by the same letter are not significantly different at the P<0.05 level.

**Fig 1:**
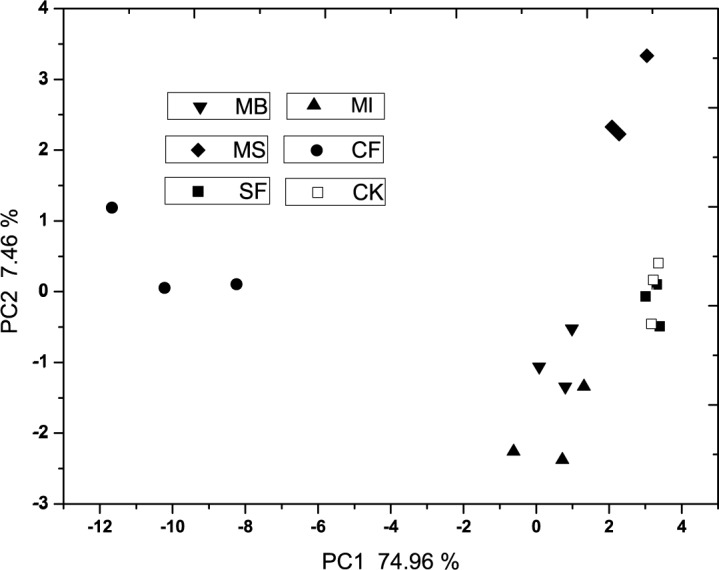
The principal component analysis (PCA) of substrates utilization patterns in fumigated treatments. MB: methyl bromide, SF: sulfuryl fluoride, MI: methyl iodide, MS: metham sodium, CF: chloroform, CK: untreated control.

**Fig 2:**
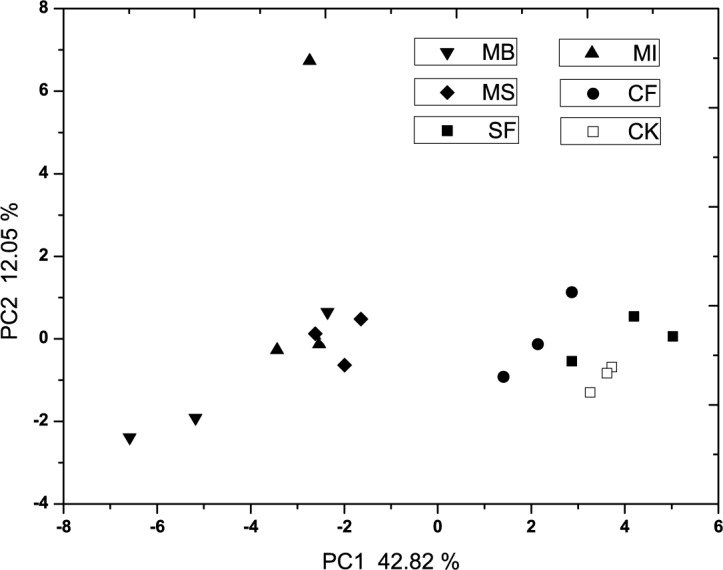
The principal component analysis (PCA) of PLFA patterns in fumigated treatments. MB: methyl bromide, SF: sulfuryl fluoride, MI: methyl iodide, MS: metham sodium, CF: chloroform, CK: untreated control.

The results of PCA of the substrates utilization patterns are shown in Fig. 1, the fumigation treatments were separated from the first principal component (PC1 = 74.96%) and the second principal component (PC2 = 7.46%). The plots of MS and CF treatments significant differed from other treatments in the axes. SF and untreated control treatment had similar PC1 and PC2 scores, there was no significant difference (p<0.05) between MB and MI in PC1 scores. In Fig. 2, the result of PCA of the PLFA patterns indicated that PC1 (42.82%) and PC2 (12.05%) explained 54.87% of the variation and discriminated among fumigation treatments. The MB treatment was significantly different (p<0.05) to the MS treatment in PC1 scores, however, MI had no significant difference with MB and MS in PC1 scores. There was no significant difference (p<0.05) between SF, CF treatment and untreated control in PC1 scores. According to the significance studies in PC2 scores, there was no significant difference (p<0.05) among all the treatments.

**Fig 3:**
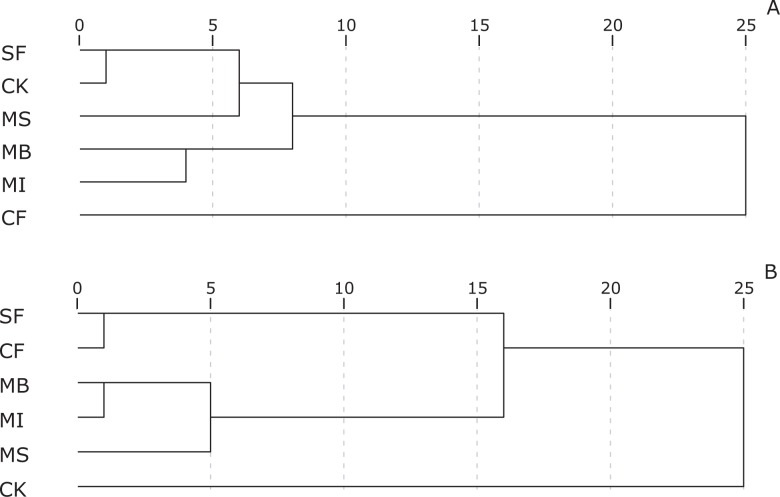
Cluster analyses of the transformed data sets derived from PLFA and Biolog analyses. A: data sets derived from Biolog analyses; B: data sets derived from PLFA analyses; Cluster methods: Ward method; Scale indicates Euclidean distance.MB: methyl bromide, SF: sulfuryl fluoride, MI: methyl iodide, MS: metham sodium, CF: chloroform,CK:untreated control.

Cluster analyses showed more visual results about influences of fumigants on soil microbial community diversities. Cluster analysis based on Biolog data sets revealed that fumigation could considerably affect the soil microbial community level physiological profiling (Fig. 3A), the SF treatment and untreated control were clustered at the shortest Euclidean distance, and CF clustered with other treatments at the farthest distance. Cluster analysis for 30 fatty acids data revealed that, initially, the SF treatment clustered with the CF treatment, then with a new branch was constructed by MB, MI and MS, and finally with untreated control (Fig. 3B). MB treatment clustered with MI treatment first in both graphs.

## Discussion

Microcosm studies indicated that MB, MI and MS could significantly control soil-borne pathogens and nematodes, thus, MI and MS can be used as potential MB alternatives for the suppression of soil-borne diseases. Many other studies are consistent with our results, Becker [11] reported that MI was more active than MB against three nematodes species (*Meloidogyne incognita*, *Heterodera schachtii*, *Tylenchulus semipenetrans*) and *R*. *solani*. Hutchinson reported that MI was 2.7 more efficacious than MB averaged over all fungal species [2]. Moreover, Noling and Gilreath [29] reported that MI rates as low as 5.6 g m^-2^ (broadcast) could provide acceptable nematode control and crop yield response. MS is a fumigant of controversial efficacy against soil-borne pathogens [30,31]. The primary breakdown product of MS in soil is methyl isothiocyanate (MITC), an active pesticidal agent with a high toxicity and a great potential for volatilization [32]. In addition, the conversion of MS in soils can be very rapid and complete within 0.5h. Other results confirmed this statement, suggesting that MS degradation to MITC was complete within 4 h. MITC decomposed quickly in a few days, except in soil containing high organic matter where it was still present after 15 days. Therefore, the effects of MS mostly depend on retaining MITC in the soil for a sufficient period of time, and obtaining a uniform distribution of MITC throughout the soil profile. MS has poor penetration ability in soil compared with MB, so a thorough mixing of the soil is necessary to ensure an even distribution and avoid phytotoxicity induced by excessive concentrations of MITC [33]. Although SF has good efficacy against a number of stored-product beetle and moth species [10,34], 33.04 mg kg^-1^ SF did not show good efficacy on soil-borne pathogens and nematodes. CF has been extensively used to determine total soil microbial biomass [35] due to its high toxicity in microbe assays. However, its high efficacy against soil microbes was not observed in our experiment, and this may be due to its low activity at normal pressure and temperature.

In keeping with their significant effect on soil-borne diseases, MB, MI and MS also significantly influenced culturable fungi, bacteria and actinobacteria, as well as soil microbial biomass carbon and nitrogen. The total amount of phospholipids is a good indicator of the living microbial biomass since the fatty acids of dead microbes are degraded immediately [36]. Results showed that MB and MI significantly decreased the total PLFA, followed by MS. The comparable results in studies on soil-borne diseases, different soil microbial populations and soil microbial biomass might be due to the same mechanism of fumigants on soil microbes. Other previous studies have also indicated similar results; for example Stromberger et al. reported that soil microbial respiration, enzyme activity, and potential nitrification rates were decreased by fumigant application, indicating a significant impact of the fumigants on the microbial flora and fauna [37]. Klose et al also indicated that among the alternative fumigants studied, MITC had the least impact on the microbial community structure[14]. In contrast to biofumigation with broccoli residue, Omirou et al found MS resulted in a more dramatic change of microbial communities. Moreover, fumigant also caused great effect on many functional microorganisms, such as methanotrophic bacteria, ammonia oxidizing bacteria[38,39].

In Biolog studies, Shannon index (an indicator of soil microbial richness and evenness), Simpson index (reflecting the abundance of the most common species), McIntosh index (a measure of uniformity) were decreased by MB, MI and MS fumigation. The fumigants which significantly affected soil microbial populations and biomass also significantly influenced the functional diversity of the microbial community. Fumigation with MB is known to result in temporary decline of bacterial species in soil [40], and use of MI also produces no long-term adverse effects on soil microbial communities [40,41]. The microbial communities can recolonize rapidly in high numbers after fumigation. However, Shannon index analysis of 30 fatty acids contradicted the Biolog results, revealing no significant difference following fumigation. This phenomenon might be explained by the heterotrophic microbes which consisted of different fatty acids using the same carbon source as their nutrition.

PCA results of the variation and discrimination among treatments based on the PLFA data sets mostly coincided to the results base on Biolog data sets. CF and MS greatly influenced the soil microbial functional diversity in the soil. MB and MI treatments greatly influenced the soil microbial community functional and structural diversity, and their effectiveness may be comparable to each other, whereas SF treatment had limited influence on the soil microbial community functional and structural diversity. SF is a broad-spectrum postharvest fumigant and first used as soil fumigant in greenhouses in China [42]. Efficacy of SF fumigation is greater against root-knot nematodes than soil-borne fungi, which could explain little influence on the soil microbial communities. Cluster analysis studies of Biolog and PLFA data sets confirmed the results showed in PCA studies, and could directly reflect information about the influences of fumigants on microbial functional and structural diversity.

In summary, fumigants with high effectiveness in suppressing soil-borne disease would significantly influence the soil microbial community in terms of soil microbial populations, biomass and diversity. Since high effectiveness against soil pathogens is accompanied by high sensitivity of microorganisms, further studies could be performed to investigate the recovery of soil microorganisms after fumigation or the addition of beneficial microorganisms to enhance the soil health after fumigation.
